# Effect of Bronchoscopist-Directed Sedation and Other Factors on Patient Comfort during Diagnostic Flexible Bronchoscopy

**DOI:** 10.1155/2022/8643844

**Published:** 2022-01-21

**Authors:** Rahul Magazine, Keerthi Nedumala Sisupalan, Vyshak Uddur Surendra, Bharti Chogtu, Preetam Rajgopal Acharya, Vasudeva Guddattu

**Affiliations:** ^1^Department of Respiratory Medicine, Kasturba Medical College, Manipal Academy of Higher Education, Manipal, Karnataka 576104, India; ^2^Department of Pharmacology, Kasturba Medical College, Manipal Academy of Higher Education, Manipal, Karnataka 576104, India; ^3^Department of Respiratory Medicine, Kasturba Medical College, Manipal Academy of Higher Education, Mangalore, Karnataka, India; ^4^Department of Data Science, Prasanna School of Public Health, Manipal Academy of Higher Education, Manipal, Karnataka 576104, India

## Abstract

**Background:**

The factors affecting patient comfort during flexible bronchoscopy are not sufficiently addressed in resource-limited countries, and a need to devise strategies to improve patient experience is felt. The present study was undertaken to assess the effect of sedation and other factors on patient comfort during flexible bronchoscopy.

**Methods:**

A total of 374 patients, aged ≥18 years undergoing flexible bronchoscopy, were enrolled in this prospective, single-center, cross-sectional study. The baseline clinical data of sedation and nonsedation groups were recorded. Anxiety level was assessed using Visual Analog Scale (VAS). Postprocedure VAS score (primary outcome measure) assessed the discomfort related to flexible bronchoscopy. Patient-reported willingness for a repeat procedure and occurrence of adverse events were used as secondary outcome measures. Based on the median of VAS score, the study population was divided into low discomfort and high discomfort groups, and the factors affecting comfort levels in these two groups were noted.

**Results:**

Postprocedural VAS score (median (Q1, Q3)) for sedation and nonsedation groups was 20 (15, 30) and 25 (20, 40), respectively (<0.001). Willingness for a repeat procedure was significantly more in the sedation group (*p*=0.002). In the low and high discomfort groups, the preprocedural anxiety level (median (Q1, Q3)) was 20 (10, 25) and 30 (20, 40), respectively (*p* < 0.001).

**Conclusion:**

Bronchoscopist-directed sedation significantly reduces patient discomfort and increases their willingness for a repeat procedure. Midazolam-fentanyl combination and lower prebronchoscopy anxiety are associated with lower discomfort levels during flexible bronchoscopy. This study is registered with the Clinical Trial Registry of India (CTRI/2018/11/016328).

## 1. Introduction

Flexible bronchoscopy, a commonly performed procedure in a pulmonary medicine unit, provides vital input by allowing direct observation of the airway and acquiring biological material for a comprehensive evaluation of the patients' clinical condition. The use of sedation during this procedure is increasingly becoming the standard of care due to the expectations of the patient to experience a comfortable procedure [1, 2]. Having said that, the majority (59.4%) of bronchoscopists in developing countries do not use sedation during flexible bronchoscopy, which contrasts with the Western world where 4–10% routinely use sedation to enhance patient comfort [3, 4]. There could be multiple reasons, such as lack of experience in use of sedatives, fear of complications arising due to sedation, and so on, for such underutilization of sedation during flexible bronchoscopy [3, 5]. Although sedatives have a major impact on the level of comfort during flexible bronchoscopy, there are other factors that can influence it as well [5–7]. Being aware of these factors can help the bronchoscopist to devise interventions to address these more effectively, thus improving the comfort level of the patient during flexible bronchoscopy. There is a pressing need to generate data from our country regarding the benefits and risks of bronchoscopist-directed conscious sedation and to understand the factors which impact patients' level of comfort during flexible bronchoscopy. With this background, the present study was conducted to assess the effect of bronchoscopist-directed conscious sedation on patient comfort during flexible bronchoscopy and to study the factors affecting patient comfort during flexible bronchoscopy.

## 2. Materials and Methods

### 2.1. Setting

**Figure 1:**
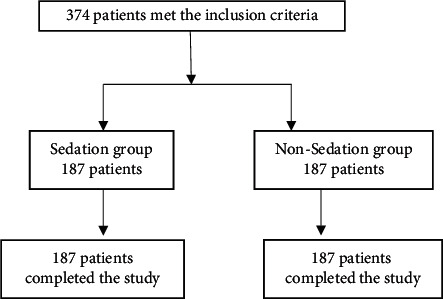
Consort diagram showing patient allocation to two groups.

A prospective, single-center, cross-sectional study was conducted at a tertiary care teaching hospital in South India after obtaining approval from the institutional ethics committee. After taking the informed consent, a total of 374 patients, who met the inclusion criteria for the study, were enrolled from December 2018 to October 2020. Consecutive eligible study subjects were allocated to one of the two groups, the sedation group or nonsedation group, based on whether they accepted bronchoscopist-directed sedation during flexible bronchoscopy or not (Figure 1). Written informed consent was obtained from the study participants after they read the patient information sheet about the study, which had been provided to them. Inclusion criterion included all patients aged ≥18 years undergoing flexible bronchoscopy in the Department of Respiratory Medicine. Patients who had undergone flexible bronchoscopy previously, patients on mechanical ventilation, patients who had a tracheostomy in place, patients having psychiatric disorders, and patients with any difficulty in communication that compromised their ability to answer questions were excluded from the study.

A prebronchoscopy questionnaire was administered to note the baseline clinical data and patients were asked to rate their anxiety level before flexible bronchoscopy using Visual Analog Scale (VAS). This was completed 12 hours prior to flexible bronchoscopy for all enrolled patients.

VAS is a psychometric response scale, which measures the patients' subjective feeling, and it was used to assess patients' anxiety prior to flexible bronchoscopy on a 10 cm horizontal line [7]. A good correlation has been noted between patients' VAS and blinded observer's VAS [8]. The left end (0 cm) of the horizontal line indicates “no anxiety,” and the right end (10 cm) indicating “severe anxiety.” The patients were asked to mark on a 10 cm horizontal line at the point corresponding to their subjective degree of anxiety before flexible bronchoscopy. VAS score was determined by measuring the distance from the left end to the mark made by the patients.

During the procedure, all patients were in the supine position. Supplemental oxygen was administered to all patients. The oxygen was titrated to keep (oxygen saturation) SpO_2_ ≥95%. Bronchoscopist-directed conscious sedation was administered if the patient had indicated a prior willingness for its use; otherwise, flexible bronchoscopy was done without sedation. The sedatives used were chosen by the bronchoscopists, and their dosage titrated based on the patients' clinical parameters—their comfort level and vitals—during the procedure. After local application of lignocaine, the flexible bronchoscopy was inserted through the nostril to reach the oropharynx. If the nasal route was not technically feasible, then the oral route was opted. Two ml of topical 2% lignocaine was sprayed on the vocal cords through the flexible bronchoscopy.

One minute later, the bronchoscope was negotiated across the vocal cords, one ml of topical 2% lignocaine was sprayed over the trachea, and one ml each into the right and left main bronchi near the carina. Once the cough subsided, the flexible bronchoscope was advanced into both bronchi for the examination of the entire bronchial tree and for carrying out the sampling procedure planned for the patient. If the patient developed a recurrent cough, an additional 2% lignocaine spray was used in aliquots of one ml at a time. Duration of the procedure was noted from the time of scope insertion to the time of removal of scope. The total lignocaine dose used for the procedure was also recorded.

After the procedure (2 hours later), again a 10 cm Visual Analogue Scale (VAS) was used to assess the patients' subjective degree of discomfort during flexible bronchoscopy. After 8 hours, patients were asked about their willingness to return for a repeat procedure if needed. For this, the patients were asked to choose from the following five options: absolutely no, probably no, I do not know, probably yes, or absolutely yes.

### 2.2. Outcome Measures

Patient-marked VAS score for assessing discomfort related to flexible bronchoscopy was used as a primary outcome measure. The discomfort felt by patients in the sedation and nonsedation groups were assessed using this score. The median of the scores of the whole study population (*N* = 374) was used to divide it into low discomfort and high discomfort groups [7]. The factors that affected comfort levels were noted based on this division. Patient-reported willingness for a repeat procedure and occurrence of adverse events in sedation and nonsedation groups were used as secondary outcome measures.

### 2.3. Statistical Analysis

The SPSS version 25.0 (SPSS Inc., Chicago, IL, USA) was used for statistical analysis. Frequency and percentage were used to summarize categorical variables. Mean and standard deviation were used to summarize normally distributed continuous variables. Median and interquartile range was used to summarize postbronchoscopy VAS score. Chi-square test was used for finding the association between two categorical variables, and independent *t*-test was used to compare the mean of continuous variables across two groups (sedated vs. nonsedated, high discomfort vs. low discomfort). Mann–Whitney *U*-test was used to compare median of nonnormally distributed variables across two groups. *p* value of <0.05 was considered as statistically significant.

### 2.4. Sample Size estimation

For a power of 80% at 95% confidence interval, a minimum of 187 patients, each in the sedation and nonsedation group, were recruited for the study.

## 3. Results

A total of 374 patients were included in the study, with 187 patients each in the sedation and nonsedation group. The baseline characteristics of each group are summarized in Table 1.

The mean duration of the procedure in the sedation and nonsedation group was 14.25 ± 5.43 and 13.87 ± 5.12 minutes (*p*=0.487). The lignocaine dose (mean ± SD) used was 185.88 mg ± 12.68 and 191.98 mg ± 19.94, respectively (*p* < 0.001). Postprocedural discomfort score and patient-reported willingness for a repeat procedure in the sedation and nonsedation group are shown in Table 2.

In the sedation group, the adverse events were as follows: uneventful (146 (78.1%)), hemoptysis (16 (8.6%)), desaturations not requiring interventions/termination of procedure (19 (10.2%)), desaturations requiring interventions (2 (1.1%)), tachycardia (2 (1.1%)), hypotension (0), bronchospasm (1 (0.5%)), and bleeding (1 (0.5%)). In the nonsedation group, the adverse events were as follows: uneventful (158 (84.5)), hemoptysis (14(7.5%)), desaturations not requiring interventions/termination of procedure (8(4.3%)), desaturations requiring interventions (2 (1.1%)), tachycardia (2(1.1%)), hypotension (1(0.5%)), bronchospasm (0), and bleeding (2(1.1%)). There was no significant difference between the two groups so far as the occurrence of adverse events was concerned (*p*=0.386).

The median VAS score of the level of discomfort related to flexible bronchoscopy was 25 mm. The patients were divided into two groups based on this median of the VAS score: low discomfort (<25 VAS) and high discomfort (≥25 VAS). Out of a total of 374 cases, 170 patients were in the low discomfort group and 204 patients in the high discomfort group. Factors affecting patient comfort during flexible bronchoscopy are summarized in Table 3. Factors affecting patient comfort in the sedation and nonsedation groups during flexible bronchoscopy are summarized in Table 4. The median of the preprocedure anxiety VAS score was 23. Patients were divided into low anxiety and high anxiety levels based on the median for the preprocedure anxiety. The mean age in years (±SD) in the low anxiety and high anxiety groups was 51.13 ± 14.38 and 54.77 ± 13.78 (*p*=0.013).

## 4. Discussion

The introduction of newer technologies and techniques has enhanced the utility of diagnostic flexible bronchoscopy but at the same time has made it a much more complex and time-consuming procedure than before. This can have adverse consequences on both the patients' comfort level as well as bronchoscopists' ease of doing the procedure. The use of conscious sedation during flexible bronchoscopy is mainly directed at improving these two aspects of the procedure; in addition, it can also have a favorable impact on the bronchoscopy-induced sympathetic response [5, 9]. Although enhancing patient comfort during flexible bronchoscopy is a need that all bronchoscopists agree with, yet there is a debate on whether patient comfort should be given precedence or the successful completion of the procedure should be the primary goal [10]. The debate may rage on, but the fact of the matter is that both these aspects need to be taken care of simultaneously. Optimizing the factors of which sedation is an important one that affect patient comfort has the potential to create favorable conditions for successful completion of the procedure as well. The assessment of patient's comfort after the completion of flexible bronchoscopy can help in improving the protocols, which bronchoscopists adopt for mitigating discomfort during future procedures. Even guidelines suggest that along with the diagnostic usefulness of flexible bronchoscopy, patient's comfort and satisfaction is an important outcome measure of the procedure [1].

Inadequate sedation is known to increase patient discomfort and reduce the willingness of the patient to return for a repeat procedure [11, 12]. In our study also, we found that postprocedure VAS score was significantly higher, indicating more discomfort, in nonsedation group when compared to the sedation group. In the flexible bronchoscopy and other clinical settings as well, the patient satisfaction determines patients' willingness to return; thus, willingness to return has been used as a surrogate indicator of patient comfort [2, 13]. The willingness to return for repeat flexible bronchoscopy varies greatly (25.1% to 93%) in different studies [2, 12, 14]. Higher percentages were noted in studies conducted in developed countries like Japan and the United States of America, whereas lower percentages were reported from developing countries like Egypt. In our study, only 29.6% in the sedation group were willing for a repeat procedure. In spite of this lower percentage of willingness for a repeat procedure in our sedation group, bronchoscopist-directed conscious sedation did increase the willingness when compared to the nonsedation group (19.3%). In developed countries, more robust protocols aimed at patient's comfort are employed, which could explain the higher percentage of patients willing for a repeat procedure in their studies. Class of sedatives chosen, the number of drugs used, their dosage, and the manner of administration over time (fixed dose or titrating doses) can affect the level of sedation and thus influence the patient comfort achieved [15].

In various studies, different sedatives—midazolam, fentanyl, propofol, dexmedetomidine, and so on—have been tried and found to improve patient comfort during flexible bronchoscopy [16–21]. The data from the present study suggest that the midazolam-fentanyl combination has a significant impact on reducing patient discomfort. A similar inference was drawn from a randomized, double-blinded, placebo-controlled Indian study where midazolam alone was compared head to head with midazolam-fentanyl combination. In this study, both midazolam and the midazolam-fentanyl combination was better than placebo, and in addition the midazolam-fentanyl combination was better than midazolam alone [16]. However, a survey from India showed that only 26.7% of those who used sedation preferred midazolam-fentanyl combination, whereas 58.3% preferred midazolam alone. Given the fact that less than half of the bronchoscopists in India use sedation at all, it is obvious that a very small percentage of patients must be receiving adequate sedation using the midazolam-fentanyl combination that is indeed a better option, if no contraindications exist [3]. In contrast to this, in Australia and New Zealand, a combination of two sedatives were used by 94%, and out of these, 96% used the midazolam-fentanyl combination [18].

The safety profile of various sedatives in common use, when compared with placebo, has been well established, and whatever complications do arise are quite manageable [16, 17, 22, 23]. Our study also found that the adverse event profile was comparable in both the sedation and nonsedation groups (*p*=0.386).

For many patients, the thought of undergoing a diagnostic flexible bronchoscopy can be a cause of high anxiety, and this preprocedure anxiety has been documented to be one of the factors associated with increased patient discomfort during flexible bronchoscopy [24, 25]. In the present study also, we found that there was a significant association between higher preprocedure anxiety and higher discomfort experienced during flexible bronchoscopy. Similar findings were found in various studies, and it has been suggested that preprocedure anxiety levels should be routinely assessed using a questionnaire and appropriate measures, like increased sedation, instituted to tackle the expected increase in discomfort among such patients [6, 7]. Interestingly, in one study, touch and verbal empathy employed prior to flexible bronchoscopy were found to reduce anxiety in patients with heightened anxiety levels [26]. On the other hand, providing detailed risk information before flexible bronchoscopy, which is a legal and ethical requirement as well, may result in marginal but significant increase in anxiety levels [27]. In our study, we found higher age to be associated with increased preprocedure anxiety, but since the difference in the mean ages is small, it does not appear to be a clinically significant difference.

While some studies have shown that the experience of the bronchoscopist influences patients' comfort levels, there are others which did not find such an association [6, 7]. In our study also, we did not note any impact of bronchoscopist experience on patient comfort level. This difference among various studies could be due to the different definitions used for a well-experienced bronchoscopist. In addition, there are subtle variables like the quality of training, which may vary among different training centers, and thus influence patient comfort. The total number of bronchoscopies done by an operator might be a better measure of expertise than a number of years of experience.

Male gender and shorter procedure time have been associated with better patient satisfaction [12]. In our study, the whole study population taken together did not reveal any association of patient discomfort with gender or procedure time, but in the sedation group, male gender was associated with lower discomfort.

### 4.1. Limitations of the Study

The outcome measure used, namely, the VAS score, is a subjective assessment tool and thus can be influenced by the personal characteristics of the patient. Since randomization was not part of the study design, a more balanced distribution of patients between the sedation and nonsedation groups could have got compromised. It is conceivable that one group could have got more such patients who are highly sensitive to even minor discomfort, thus affecting the results. Nevertheless, patient marked VAS is a commonly used tool with its validity backed by a decent amount of evidence, and good correlation noted with a blinded observer marked VAS [8, 28]. Other factors that can potentially influence patient comfort levels, such as time spent by the bronchoscopist in explaining the procedure, were not assessed. Also, the clinician's satisfaction with the procedure, which has been assessed in similar studies, was not captured [8]. Patient's satisfaction with sedation instrument and clinician's satisfaction with sedation instrument are reliable and validated tools for gastrointestinal endoscopy, and these could be used as well. Despite being potentially useful, these have not been used much in the context of bronchoscopy [29].

## 5. Conclusion

Bronchoscopist-directed conscious sedation significantly reduces the patient's discomfort and increases their willingness for a repeat procedure. Lower prebronchoscopy anxiety levels are associated with lower discomfort levels during flexible bronchoscopy. Midazolam-fentanyl combination is associated with significantly lower levels of discomfort during flexible bronchoscopy. Midazolam used alone also showed a similar trend although not reaching statistical significance.

## Figures and Tables

**Table 1:** Demographic characteristics of patients in sedation and nonsedation groups (*n* = 374).

Characteristics	Sedation (*n* = 187)	Nonsedation (*n* = 187)	*p*-value
Age (years) (mean ± SD)	51.64 ± 14.06	54.29 ± 14.23	0.071^*∗*^
Male/female	107/80	115/72	0.40^#^
Weight in kg (mean ± SD)	52.71 ± 11.06	51.84 ± 9.57	0.418^*∗*^
Height in cm (mean ± SD)	158.06 ± 6.1	158.57 ± 6.28	0.481^*∗*^
BMI kg/m^2^(mean ± SD)	21.02 ± 3.83	20.58 ± 3.28	0.239^*∗*^
Comorbidities, *N* (%)	126 (67.0)	117 (62.6)	0.329^#^
*Indication for bronchoscopy*			
Tubercular infection	75 (40.1)	73 (39.0)	0.973^#^
Nontubercular infection	69 (36.9)	71 (38.0)	
Malignancy	43 (23.0)	43 (23.0)	
Preprocedural anxiety level (VAS score) (median (Q1, Q3)	20 (15, 35)	25 (15, 35)	0.996^*∗∗*^
Route of bronchoscope insertion			
Transnasal	173 (92.5)	168 (89.8)	0.831^#^
Transoral	14 (7.5)	19 (10.2)	
*Bronchoscopic procedure performed*			
Bronchial lavage	187 (50%)	187 (50%)	—
Biopsy/TBNA	24 (12.8%)	19 (10.2%)	0.418^#^
Bronchial brushing	40 (21.4%)	47 (24.6%)	0.461^#^

^#^Chi-square test. ^*∗*^Independent sample *t*-test. ^*∗∗*^Mann–Whitney *U* test. TBNA: transbronchial needle aspiration.

**Table 2:** Postflexible bronchoscopy outcome measures.

Outcome measures	Sedation (*n* = 187)	Nonsedation (*n* = 187)	*p*-value
Postprocedural discomfort score (VAS score) (median (Q1, Q3)	20 (15,30)	25 (20,40)	<0.001^*∗∗*^
Patient-reported willingness for a repeat procedure, if required in the future			
Absolutely no	15 (8)	37 (19.8)	0.002^#^
Probably no	33 (17.6)	43 (23)	
I do not know	84 (44.9)	71 (38)	
Probably yes	52 (27.8)	31 (16.6)	
Absolutely yes	3 (1.6)	5 (2.7)	

^#^Chi-square test. ^*∗∗*^Mann–Whitney *U* test.

**Table 3:** Factors affecting patient comfort during flexible bronchoscopy.

Characteristics	Low discomfort VAS <25, *n* = 170	High discomfort VAS ≥25, *n* = 204	*p*-value
Age (mean years ± SD)	52.00 ± 14.54	53.76 ± 13.87	0.232^*∗*^
Male: female	108 : 62	114 : 90	0.134^#^
Addictions, *n* (%)			
Smokers (including ex-smokers)	34 (20.0)	51 (25.0)	0.120^#^
Alcoholics	2 (1.2)	6 (2.9)	
Both (smokers and alcoholics)	8 (4.7)	3 (1.5)	
Nil addictions	126 (74.1)	144 (70.6)	
Any comorbidities, *n* (%)	108 (63.53)	135 (66.2)	0.593^#^
Socioeconomic class, *n* (%)			
Lower class	2 (1.2)	4 (2)	0.674^#^
Upper lower class	36 (21.2)	49 (24.0)	
Lower middle class	97 (57.1)	116 (56.9)	
Upper middle class	34 (20.0)	32 (15.7)	
Upper class	1 (0.6)	3 (1.5)	
Indication for flexible bronchoscopy, *n* (%)			
Tubercular	69 (40.6)	79 (38.7)	0.865^#^
Other infections	64 (37.6)	76 (37.3)	
Malignancy	37 (21.8)	49 (24.0)	
Preprocedural anxiety level (VAS score) (median (Q1, Q3)	20 (10, 25)	30 (20, 40)	*<0.001* ^*∗∗*^
Route of bronchoscope insertion, *n* (%)			
Transnasal	155 (91.2)	186 (91.2)	1.000^#^
Transoral	15 (8.8)	18 (8.8)	
Midazolam used, *n* (%)	58 (34.1)	54 (26.5)	0.068^#^
Midazolam not used, *n* (%)	112 (65.9)	150 (73.5)	
Midazolam fentanyl combination used, *n* (%)	35 (20.6)	22 (10.8)	0.009^#^
Midazolam fentanyl combination not used, *n* (%)	135 (79.4)	182 (89.2)	
Lignocaine dose (mean ± SD)	187.65 ± 15.55	190.0 ± 18.03	0.182^*∗*^
Bronchoscopic interventions done, *n* (%)			
Bronchial lavage	170 (100)	204 (100)	—
Biopsy/TBNA	21 (12.4)	22 (10.8)	0.636^#^
Bronchial brushing	41 (24.1)	45 (22.1)	0.638^#^
*Experience of the operator*			
Less experienced (0–4 years)	125 (73.5)	141 (69.1)	0.349^#^
More experienced (>4 years)	45 (26.5)	63 (30.9)	
Duration of procedure in minutes (mean ± SD)	14.10 ± 4.87	14.02 ± 5.6	0.884^*∗*^
Procedure-related adverse events during or in the immediate postprocedure period	35 (20.6)	36 (17.6)	0.470^#^

^#^Chi-square test. ^*∗*^Independent Student's *t*-test. ^*∗∗*^Mann–Whitney *U* test.

**Table 4:** Factors affecting patient comfort in sedation and nonsedation groups during flexible bronchoscopy.

Characteristics	Sedation (*N* = 187)			Nonsedation (*N* = 187)		
	Low discomfort VAS <25, *n* = 103	High discomfort VAS ≥25, *n* = 84	*p*-value	Low discomfort VAS <25, *n* = 67	High discomfort VAS ≥25, *n* = 120	*p*-value
Age (mean years ± SD)	50.30 ± 14.05	53.27 ± 13.98	0.368^*∗*^	54.61 ± 15.06	54.11 ± 13.83	0.637^*∗*^
Male: female	66 : 37	41 : 43	0.036^#^	42 : 25	73 : 47	0.803^#^
Addictions, *n* (%)						
Smokers (including ex-smokers)	17 (16.5)	18 (21.4)	0.303^#^	17 (25.4)	33 (27.5)	0.311^#^
Alcoholics	2 (1.9)	2 (2.4)		0 (0)	4 (3.3)	
Both (smokers and alcoholics)	4 (3.9)	0 (0)		4 (6.0)	3 (2.5)	
Nil addictions	80 (77.7)	64 (76.2)		46 (68.7)	80 (66.7)	
Comorbidities, *n* (%)	70 (68)	56 (66.7)	0.851^#^	38 (56.7)	79 (65.8)	0.217^#^
Socioeconomic class, *n* (%)						
Lower class	2 (1.9)	0 (0)	0.480^#^	0 (0)	4 (3.3)	0.395^#^
Upper lower class	20 (19.4)	22 (26.2)		16 (23.9)	27 (22.5)	
Lower middle class	55 (53.4)	43 (51.2)		42 (62.7)	73 (60.8)	
Upper middle class	25 (24.3)	19 (22.6)		9 (13.4)	13 (10.8)	
Upper class	1 (1.0)	0 (0)		0 (0)	3 (2.5)	
*Indication for flexible bronchoscopy, n (%)*						
Tubercular	39 (37.9)	36 (42.9)	0.475^#^	30 (44.8)	43 (35.8)	0.444^#^
Other infections	42 (40.8)	27 (32.1)		22 (32.8)	49 (40.8)	
Malignancy	22 (21.4)	21 (25.0)		15 (22.4)	28 (23.3)	
Preprocedural anxiety level before examination (VAS score) (median (Q1, Q3))	15 (10,20)	35 (26,45)	<0.001^*∗∗*^	15 (10,20)	35.20 (30,45)	<0.001^*∗∗*^
Lignocaine dose (mean ± SD)	185.83 ± 12.41	185.95 ± 13.09	0.946^*∗*^	190.45 ± 19.18	192.83 ± 20.83	0.392^*∗*^
*Bronchoscopic interventions done, n (%)*						
Bronchial lavage	103 (100)	84 (100)	—	67 (100)	120 (100)	—
Biopsy/TBNA	13 (12.6)	11 (13.1)	0.923^#^	8 (11.9)	11 (9.2)	0.547^#^
Bronchial brushing	21 (20.4)	19 (22.6)	0.711^#^	20 (29.9)	26 (21.7)	0.213^#^
Duration of procedure (minutes) (mean ± SD)	13.92 ± 4.856	14.64 ± 6.07	0.151^*∗*^	14.37 ± 4.93	13.58 ± 5.22	0.952^*∗*^
Procedure-related adverse events during or in the immediate postprocedure period	23 (22.3)	18 (21.42)	0.417^#^	12 (17.91)	17 (14.16)	0.444^#^

^#^Chi-square test. ^*∗*^Independent Student's *t*-test. ^*∗∗*^Mann–Whitney *U* test.

## Data Availability

The data used to support the findings of this study are available from the corresponding author upon request.
